# Impact of successful antegrade and retrograde CTO PCI on short-term prognosis

**DOI:** 10.1186/s43044-024-00501-6

**Published:** 2024-05-31

**Authors:** Khaled Adel El Etriby, Nireen Khalifa Okasha, Mohamed El-Sayed Zahran, Tarek Rashid Mohamed

**Affiliations:** Department of Cardiology, Faculty of Medicine, Ain Shams University, Cairo, Egypt

**Keywords:** CTO, PCI, Antegrade, Retrograde, MACE, TLR

## Abstract

**Background:**

Chronic total occlusion (CTO) percutaneous coronary intervention (PCI) carries risk of complications and should be attempted when the anticipated benefits exceed the potential risks. The primary indication for CTO–PCI is symptom improvement. However, the impact of CTO–PCI on angina and subsequent incidence of major adverse cardiovascular event (MACE) rate remains controversial. Our aim was to study the impact of technically successful elective CTO–PCI on the procedural success rate and short-term MACE. The current study was a prospective cohort study that included a total of 80 patients who were referred to our center (Ain Shams University Hospitals) for elective CTO–PCI and underwent technically successful CTO–PCI. Data were collected on patient arrival to our department, and then, the patients were observed during hospital admission to record any In-Hospital MACE. These patients were then followed up for 6 months to record improvement or worsening of their symptoms and to assess occurrence of any MACE including hospitalization and undergoing symptom-driven coronary angiography.

**Results:**

The mean age of our patients was 56 ± 9.6 years, and 73 patients (91%) were men. Sixty-two patients (77.5%) were done via an antegrade approach, and 18 patients (22.5%) were done via a retrograde approach with an overall procedural success rate of 91.25% (antegrade 93.5%, retrograde 83.3%). The overall mean procedure time was 102 min, the mean contrast volume used was 371 ml, and the mean cumulative air kerma dose was 7.2 Gy. The retrograde group required longer procedure times, larger volumes of contrast and higher exposure to radiation. The overall in-hospital MACE was 8.75%. Sixty-five patients in our study (81.25%) showed an improvement in the grade of their exertional dyspnea or angina within the 6-month follow-up period. Thirteen patients in our study (16.25%) needed re-hospitalization within a 6-month period after PCI. The overall target lesion revascularization rate at 6 months was 8.75%.

**Conclusions:**

Technically successful CTO PCI in a well-equipped center with highly qualified CTO operators resulted in high procedural success rates and low incidence of short-term MACE.

## Background

CTO-PCI carries risk of complications and should be attempted when the anticipated benefits exceed the potential risks. According to a recently published global expert consensus, the primary indication for CTO-PCI is symptom improvement, as is true for PCI in all patients with stable coronary artery disease [1]. Whether CTO-PCI can reduce the risk for a subsequent major adverse cardiovascular event (MACE) remains controversial [2].

CTOs are defined as coronary obstructions which produce total occlusion of vessel lumen with thrombolysis in myocardial infarction (TIMI 0) flow and duration longer than three months [3].

The retrograde approach is currently an essential tool for achieving high success rates, especially in complex lesions where the antegrade approach is not technically feasible or fails [4].

In multicenter registries of consecutively evaluated patients undergoing first diagnostic coronary angiography because of symptoms of typical angina pectoris or dyspnea, CTO of major epicardial coronary arteries was present in up to one-third of patients [5].

The introduction of retrograde CTO crossing techniques was instrumental in increasing CTO PCI success rates from < 70% to nearly 90% [6].

Registries consistently show that antegrade wiring (AW) is the most common strategy for crossing CTOs, particularly those of lower complexity [7].

The worldwide procedural success rate of CTO-PCI has impressively improved after the introduction of dedicated devices and increased operators’ clinical experience, and the threshold for performing PCI of more complex CTO has thereby been lowered. In dedicated CTO-PCI centers, success rates of CTO-PCI have been reported of up to 95% [8].

Alongside the continuous improvement in success rates over the years, there has also been a noticeable decrease in the complications associated with these procedures [9].

## Methods

This study was a prospective cohort study, conducted from October 2021 to October 2022 at Ain shams university hospital, Cairo, Egypt. Ain shams university ethical committee has approved this study, and all participants have signed the written informed consent. Participants were oriented by the nature of the study and the data extracted from this study. Confidentiality and privacy of data were ensured to all participants. The participation was on a voluntary basis and the participants had the right to withdraw at any time.

The study population included patients with a CTO of one or more coronary arteries proved by coronary angiography and indicated for PCI according to recent European and American guidelines of coronary revascularization, patients needed to provide objective evidence of ischemia and viability via non-invasive tests to be included except in specific cases where patients who were suffering from angina CCS grade 3 or 4 and not responding to guideline directed medical treatment and patients who complained of new crescendo angina not explained by other lesions and the operator decided to intervene based on clinical data [10, 11]. All patients were required to have a technically successful CTO PCI procedure to be included in our study. Operators were not bound to use a specific algorithm for wire crossing and for choosing the primary approach for crossing whether antegrade or retrograde, it was based on the operator’s decision after reviewing all the patient’s data and previous coronary angiography and each operator would decide based on any of the available crossing algorithms mentioned in the literature. Retrograde approach was also to be used as a secondary approach in case of failure of primary antegrade approach and if successful would be included in the successful retrograde group dataset.

Participants were excluded from our study if coronary artery bypass grafting was the choice for revascularization, if they needed concomitant valvular or aortic surgery, if they suffered from a recent (less than one month) acute coronary syndrome or had overt heart failure or cardiogenic shock. They were also excluded if their life expectancy was less than 1 year as a result of non-cardiac conditions or if their creatinine clearance was less than 30 ml/hr.

## Research participants

This study included a total of 80 patients who were referred to our center for elective CTO PCI and underwent technically successful CTO PCI. All patients were recruited from the Cath-lab of the Cardiology department at Ain Shams University Hospitals.

Convenience sampling method was used to recruit the participants in our study. Data were collected on patient arrival to our department before the PCI procedure, and then, the patients were observed during the period of hospital admission to record any In-Hospital MACE.

These patients were then followed up for 6 months to record improvement or worsening of their symptoms and to assess occurrence of any major adverse cardiac event including hospitalization for angina or heart failure symptoms and undergoing symptom-driven coronary angiography.

All patients after written informed consent were subjected to the following at baseline: Obtaining a detailed clinical history with attention paid to the age and sex of the patients, presence of different comorbidities including smoking status, diabetes mellitus, hypertension, dyslipidemia, family history of premature CAD, renal impairment, heart failure, peripheral arterial disease and if they complained of any symptoms including chest pain (CCS class) and dyspnea (NYHA class), previous history of PCI or CABG and whether they had done any non-invasive imaging tests to guide the procedure such as stress echo or stress MPI or MSCT coronary angiography.

Transthoracic echocardiography was done to assess baseline Left Ventricular Ejection Fraction using multiple methods (M-mode & 2-D eye-balling). A GE vivid S5N machine with an RS3 probe were used.

Regarding procedural aspects, PCI was done according to currently recommended best practice and was performed by expert operators. CTO lesions complexity and CTO PCI attempt difficulty were evaluated based on the J-CTO score. The Japanese CTO score involved a 5-point scoring system as follows: blunt stump, calcification, bending > 45 degrees within the lesion, occlusion length 20 mm or more and prior failed attempt to revascularize the CTO.

Data collected regarding procedural characteristic included the vascular access site, CTO vessel, procedure time (min), radiation dose (cumulative AK/ Gy), number and type of wires used, use of microcatheter, contrast dose (ml), number of stents, number of balloons and crossing strategies (antegrade, retrograde). We used General Electric (GE), Philips and SIEMENS catheterization systems for our procedures.

Technical success was defined as final residual stenosis < 30% with restoration of TIMI flow grade 3 at the end of the procedure. Procedural success was defined as technical success without in-hospital cardiac events such as death, myocardial infarction or injury, urgent coronary artery bypass grafting (CABG), or urgent repeat PCI.

Post-procedure, medications were prescribed according to 2018 ESC myocardial revascularization guidelines [10]. Serial cardiac troponin was measured within the first 6–24 h after the procedure. Patients were assessed for immediate adverse outcomes (Perforation with or without tamponade, vascular access complications, myocardial infarction or injury (Peri-procedural myocardial injury was defined as an increase of at least one cTn value > 99th percentile URL) [12], urgent coronary artery bypass grafting, urgent repeat PCI or death).

Patients were followed up for 6 months to assess occurrence of any component of MACE (death from any cause, non-procedural myocardial infarction, target lesion revascularization (TLR), target vessel revascularization (TVR) or stroke) and to record improvement or recurrence of symptoms (chest pain (CCS class) and dyspnea (NYHA class)). Symptomatic patients who underwent post-procedural symptom-driven coronary angiography, their angiographic results were assessed regarding presence of TLR, TVR or de novo lesion.

### Statistical analysis

Data were coded and entered using SPSS program version 20. Suitable statistical tests will be used. Quantitative data were expressed as the mean + standard deviation (SD). Unpaired t-test was used to compare between 2 groups as regards quantitative variables. Chi-square test was used to compare qualitative variables. A value of *P* < 0.05 was considered statistically significant. There were no procedures with missing data in any important field, and no cases had any missing outcome data. Therefore, all cases were analyzed for outcome and no missing data biased this study.

## Results

During the study period from October 2021 to October 2022, patients were referred to our center for elective CTO PCI and 80 of them were included after having a technically successful CTO PCI procedure. Our primary end point was to detect occurrence of any In-hospital MACE to assess the overall procedural success rate. Secondary end points included follow-up data collected within 6 months to record improvement or worsening of patient’s symptoms and to assess occurrence of any of the individual major adverse cardiac events.

As seen in Table 1, the mean age of the patients was 56 ± 9.6 years old, 91% of patients were males, 52% were hypertensive, 53% were diabetic, 63% were current or ex-smokers, 72% were dyslipidemic, and 20% had a previous family history of IHD. 66% of patients complained of exertional dyspnea pre-PCI, and 56% complained of exertional angina. Forty-eight % of patients had underwent previous PCI, and 10% were post-CABG.

**Table 1:** Demographic data of all participating patients

Age	Range	29	−	78
	Mean ± SD	56.038	±	9.613
		*N*		%
Sex	Male	73		91.25
	Female	7		8.75
Hypertension		42		52.50
DM		43		53.75
Smoker		51		63.75
Dyslipidemia		58		72.50
Family history of ischemic heart disease		16		20.00
Dyspnea before PCI		53		66.25
Dyspnea NYHA class before PCI	G2	27		50.94
	G3	26		49.06
Angina before PCI		45		56.25
Angina CCS class before PCI	G2	26		57.78
	G3	19		42.22
Previous PCI		39		48.75
Post-CABG		8		10.00

*NYHA* New York Heart Association, *CCS* Canadian Cardiovascular Society, DM diabetes mellitus, PCI percutaneous coronary intervention, CABG coronary artery bypass graft surgery

As seen in Table 2, 96% of patients had only 1 vessel with chronic total occlusion and 4% had 2 vessels with CTO. Regarding the vessels affected, 45% were CTO LAD, 43.75% CTO RCA and 15% CTO LCX.

**Table 2:** Showing the CTO vessel site, vascular access, the approach used and procedural success rate

Total	*N*	%
No. of CTO vessels		
One	77	96.25
Two	3	3.75
LAD	36	45.00
LCX	12	15.00
RCA	35	43.75
Vascular access site		
Rt Femoral	10	12.50
Bilateral femoral	47	58.75
Rt femoral & Rt radial	23	28.75
CTO approach		
Antegrade	62	77.50
Retrograde	18	22.50
Procedural success	73	91.25

Regarding the vascular access site, 12.5% of patients were done via right femoral access only, 58.75% bilateral femoral access and 28.75% had a right radial and right femoral access.

Seventy-seven % of patients were done via an antegrade approach, and 23% were done via a retrograde approach, all were technically successful with an overall procedural success rate of 91.25% as seen in Table 2.

The procedural success rate was higher in the antegrade group with 93.55% success, while the retrograde group had a procedural success rate of 83.33%, but this difference was statistically non-significant with a P-value of 0.177.

As seen in Table 3, the mean EF of our patients by trans-thoracic echo was 50.95%.

**Table 3:** Non-invasive tests used in planning the procedure

	*N*	%
Stress echo	45/80	56.25
Stress MPI	33/80	41.25
MSCT CA	11/80	13.75
EF (%)	Range	30–72
	Mean ± SD	50.950 ± 10.704

*MPI* myocardial perfusion imaging, *MSCT CA* multislice computed tomography coronary angiography, *EF* ejection fraction

Forty-five patients (56%) had a pharmacological stress echo done before the procedure, and 33 patients (41%) had a stress MPI test done. (Tests were done to establish both ischemia and viability).

Only 2 out of the 80 patients did not undergo a stress test pre-PCI due to the severity of their symptoms, and the operator’s decision was to proceed without the need for a non-invasive test.

Eleven patients (13.75%) had a MSCT CA done pre-PCI which included all post-CABG patients and 3 other patients where MSCT was needed to guide the procedure.

As seen in Table 4, the overall in-hospital MACE was 8.75%, 1 case needed urgent repeat PCI, another case had a cardiac arrest in the CCU a few hours after the procedure due to incessant VT followed by brady-asystole and 5 cases had peri-procedural myocardial injury.

**Table 4:** In-hospital MACE and procedural complications

Total		
	*N*	%
In-hospital MACE Total	7	8.75
Urgent repeat PCI/CABG	1	1.25
Myocardial injury	5	6.25
Death	1	1.25
Procedural complications		
Perforation without tamponade	3	3.75
Vascular access complications	2	2.50

Regarding procedural complications, there were 3 cases where perforation occurred during the procedure but without tamponade and there were only 2 cases where vascular access complications occurred.

Table 5 shows that 16.25% of patients needed hospitalization within a 6-month period after PCI. One case was admitted with STEMI (same vessel), 4 with N-STEMI and 8 cases with unstable angina. All 13 patients had a repeat coronary angiography done during hospitalization.

**Table 5:** Six-month follow-up of occurrence of any major adverse cardiac event

Six-month follow-up		
	*N*	%
No. of patients who needed hospitalization	13	16.25
Hospitalization type		
STEMI	1	7.69
NSTEMI	4	30.77
UA	8	61.54
Repeat CA	13	16.25
Target vessel revascularization (TVR)	1	7.69 (1.25% overall)
Target lesion revascularization (TLR)	7	53.85 (8.75% overall)
De novo lesion	2	15.38
Patent stents in CTO vessel	6	46.15

*STEMI* ST-elevation myocardial infarction, *NSTEMI* non-ST elevation myocardial Infarction, *UA* unstable angina

Within those 13 patients, 6 of them showed patent stents in the CTO vessel and 7 of them needed target lesion revascularization (TLR) due to significant in-stent re-stenosis. Two of the 13 cases had a de novo lesion in a different vessel to the one where PCI was done previously. One case needed target vessel revascularization due to a new lesion in the same vessel distal to the previously deployed patent stents. The overall TLR rate at 6 months was 8.75%, and the overall TVR rate at 6 months was 1.25%.

Table 6 shows that 65 patients (81.25%) had an improvement in the grade of their exertional dyspnea or angina after a 6-month period of at least 1 NYHA or CCS class.

**Table 6:** Six-month follow-up of symptoms

Six-month follow-up		
	*N*	%
Improvement of symptoms	65/80	81.25
Dyspnea After PCI	39	48.75
Dyspnea NYHA class After PCI		
G1	28	71.79
G2	6	15.38
G3	5	12.82
Angina After PCI	23	28.75
Angina CCS class After PCI		
G1	12	52.17
G2	6	26.09
G3	5	21.74

*NYHA* New York Heart Association, *CCS* Canadian Cardiovascular Society

Twenty-three patients had complete freedom from both angina and dyspnea after 6-month follow-up.

Twelve patients with previous angina had an improvement in their CCS class with all 12 reaching CCS class 1 after 6 -month follow-up.

Thirty patients with previous dyspnea had an improvement in their NYHA class with 28 of them reaching NYHA class 1, and 2 patients reached NYHA class 2 which was an improvement on their NYHA class pre-intervention.

Remaining patients did not show improvement of their symptoms and had various grades of dyspnea and angina with some reaching NYHA class 3 and CCS class 3 as seen in this table with five patients suffering from both dyspnea and angina of various grades.

Table 7 shows that the mean J-CTO score in the retrograde group was higher with a value of 3.7 compared to 2.4 in the antegrade group which was statistically significant with a P-value of less than 0.001.

**Table 7:** Comparing the J-CTO score between the antegrade and retrograde groups

	CTO approach				Chi-square	
	Antegrade		Retrograde			
	*N*	%	*N*	%	*X*^2^	*P*-value
J-CTO score blunt cap	29	46.77	13	72.22	3.623	0.057
J-CTO score calcification	27	43.55	14	77.78	6.542	0.011*
J-CTO score bend > 45	31	50.00	15	83.33	6.343	0.012*
J-CTO score length > 20 mm	51	82.26	17	94.44	1.625	0.202
J-CTO score previously failed	11	17.74	10	55.56	10.303	0.001*
J-CTO score						
Intermediate (1)	12	19.35	0	0.00	17.167	< 0.001*
Difficult (2)	22	35.48	0	0.00		
Very difficult (≥ 3)	28	45.16	18	100.00		

*T*-Test							*t*	*P*-value
J—CTO score total								
Range	1	–	5	3	–	5	− 5.666	< 0.001*
Mean ± SD	2.403	±	0.983	3.778	±	0.548		
Yes	58		93.55	15		83.33		

**Fig. 1:**
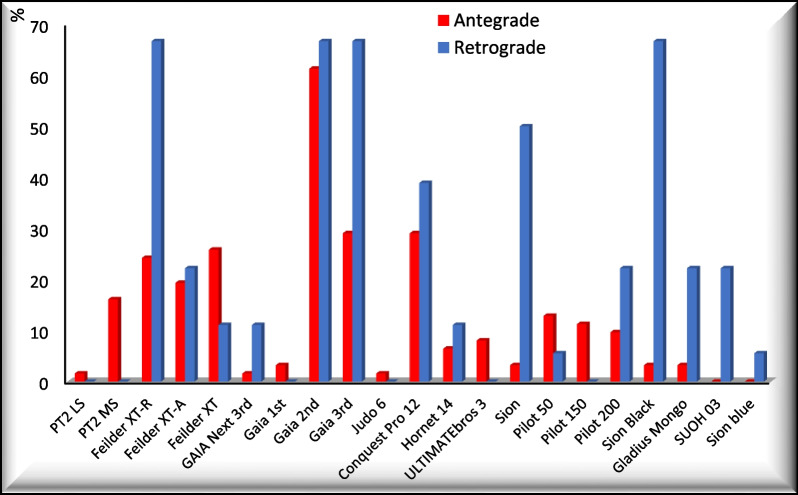
Guidewires used in the 2 groups

As seen in Fig. 1, there were many guidewires that were used in a higher percentage of cases in the antegrade group such as the Pilot 50, Pilot 150, ULTIMATEbros 3, Fielder XT and PT2-MS guide wires. There were also many guidewires that were used in a higher percentage of cases in the retrograde group due to their specific profiles which are useful in crossing collaterals or crossing a tough proximal cap such as the Fielder XT-R, Sion, Sion black, SUOH 03 and GAIA 3rd wires.

**Fig. 2:**
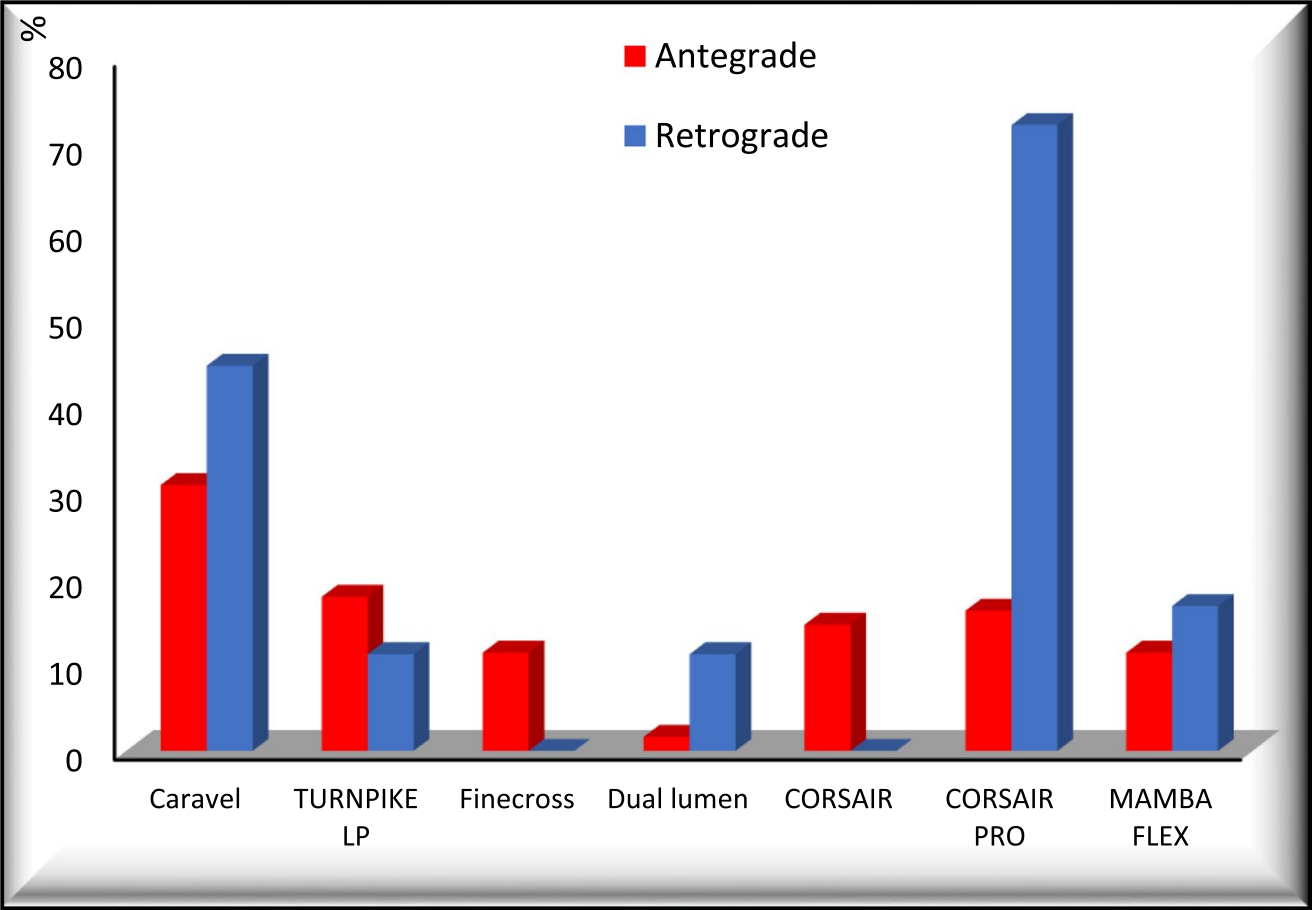
Microcatheters used in the 2 groups

Figure 2 shows that most micro-catheters had similar usage between the 2 groups except for the CORSAIR-PRO microcatheter which was used in a much higher percentage of retrograde cases compared to the antegrade group.

Table 8 shows that the retrograde group had a higher procedure time, contrast volume used and higher radiation doses compared to the antegrade group which was statistically significant with a P-value of less than 0.001.

**Table 8:** Comparing procedural details between the antegrade and retrograde groups

	CTO approach						*T*-Test	
	Antegrade			Retrograde			*t*	*P*-value
Procedure time (min.)								
Range	30	−	180	75	−	170	− 5.110	< 0.001*
Mean ± SD	92.919	±	34.430	136.778	±	21.471		
Contrast volume (ml)								
Range	150	–	550	250	–	600	− 6.120	< 0.001*
Mean ± SD	338.710	±	91.624	486.111	±	83.676		
Radiation dose cumulative AK /Gy								
Range	2.4	−	12	2.1	−	12.7	− 5.545	< 0.001*
Mean ± SD	6.437	±	2.261	9.839	±	2.398		

Table 9 shows that there was no statistically significant difference between the 2 groups regarding overall in-hospital MACE and procedural complications, but the retrograde group showed an increase in peri-procedural myocardial injury which was statistically significant with a P-value of 0.038.

**Table 9:** Comparing the in-hospital MACE and procedural complications between the 2 groups

	CTO approach				Chi-square	
	Antegrade		Retrograde			
	*N*	%	*N*	%	*X*^2^	*P*-value
In-hospital MACE total	4/62	6.45	3/18	16.67	1.823	0.177
Urgent repeat PCI/CABG	1/62	1.61	0	0.00	0.294	0.588
Myocardial injury	2/62	3.23	3/18	16.67	4.301	0.038*
Death	1/62	1.61	0	0.00	0.294	0.588
Procedural complications:						
Perforation without tamponade	1/62	1.61	2/18	11.11	3.487	0.062
Vascular access complications	1/62	1.61	1/18	5.56	0.890	0.346

Table 10 shows that a higher percentage of the antegrade group cases needed hospitalization and repeat angiography, but the difference was not statistically significant.

**Table 10:** Comparing the 6-month follow-up of occurrence of any MACE between the 2 groups

	CTO approach				Chi-square	
	Antegrade		Retrograde			
	*N*	%	*N*	%	*X*^2^	*P*-value
No. of patients who needed hospitalization	12	19.35	1	5.56	1.952	0.162
Hospitalization Type						
STEMI	1	8.33	0	0.00	2.438	0.296
NSTEMI	3	25.00	1	100.00		
UA	8	66.67	0	0.00		
Repeat CA	12	19.35	1	5.56	1.952	0.162
Target vessel revascularization (TVR)	1	8.33	0	0.00	0.090	0.764
Target lesion revascularization (TLR)	7	58.33	0	0.00	1.264	0.261
De novo lesion	1	8.33	1	100.00	5.958	0.015*
Patent stents in CTO vessel	5	41.67	1	100.00	1.264	0.261

The only case from the retrograde group that underwent repeat coronary angiography showed patent stents in the CTO vessel but showed a de novo lesion in another vessel.

Table 11 shows that 3 out of the 7 patients (42.86%) who suffered from in-hospital MACE developed another Major adverse cardiac event during the 6-month follow-up period. This was a higher percentage than the patients who did not suffer from in-hospital MACE and was statistically significant with a P value of 0.046.

**Table 11:** Correlation between the occurrence of in-hospital MACE and the occurrence of MACE during the 6-month follow-up period

Six-month MACE	In-hospital MACE				Chi-square	
	No		Yes			
	N	%	N	%	X^2^	P-value
Hospitalization and repeat CA done	10/73	13.70	3/7	42.86	3.990	0.046*
Hospitalization Type						
STEMI	1/10	10.00	0	0.00	0.325	0.850
NSTEMI	3/10	30.00	1/3	33.33		
UA	6/10	60.00	2/3	66.67		
Target vessel revascularization (TVR)	1/10	10.00	0	0	0.325	0.569
Target lesion revascularization (TLR)	6/10	60.00	1/3	33.33	0.660	0.416
De novo lesion	1/10	10.00	1/3	33.33	0.965	0.326
Patent stents in CTO vessel	4/10	40.00	2/3	66.67	0.660	0.416

Regarding these 3 patients, the first one needed urgent repeat PCI during his in-hospital stay and later on during the 6-month follow-up period, he was admitted with NSTEMI and CA showed significant ISR and required TLR.

The other 2 patients had peri-procedural myocardial injury and during the 6 -month follow-up period, they were admitted with unstable angina and CA showed patent stents but one of them had a de novo lesion in another vessel which required revascularization.

## Discussion

Chronic total occlusion (CTO) of coronary arteries represents an advanced form of atherosclerotic coronary artery disease, which is currently prevalent in almost one-fifth of patients presenting for diagnostic coronary angiography [13]. Revascularization of CTO using PCI has several clinical benefits, including ischemic symptom relief and quality of life improvement. These findings are supported to date by limited randomized controlled studies, yet it is still unclear whether revascularization provides a survival benefit or long-term freedom from cardiac events [14].

The mean age of our patients was 56 ± 9.6 years and 91% of the patients were men. The mean age seen in international registries was found to be to be around 10 years higher than our study as seen in the OPEN-CTO (65 years), RECHARGE (66 years) and EURO-CTO (62 years). This could be explained by the very high prevalence of atherosclerotic cardiovascular disease in our region and its consequences appearing at an earlier age in our patients. The very high percentage of male patients in our study is also consistent with these large registries where the percentage of males ranged from 80 to 88% [15–17].

Seventy-seven % of patients were done via an antegrade approach and 23% were done via a retrograde approach with an overall procedural success rate of 91.25% (Antegrade 93.5%, Retrograde 83.3%).

All the procedures done in this study were by expert high volume CTO operators which explains why the procedural success rate in our center was comparable to large international CTO registries such as the OPEN CTO registry [14] which has a 90% procedural success rate, the PROGRESS-CTO registry [18] with 87% and the EURO-CTO with 88% [19].

Our success rate was higher than all-comer registries where multiple operators with variable levels of experience are involved such as the NCDR (National Cardiovascular data registry) [20] in the USA which showed a 59% procedural success rate and the BCIS (British cardiovascular society) registry [21] which showed a 67% procedural success rate. Therefore, it is vital to have your most experienced CTO operators performing the procedure to maintain the high standards required in your CTO program [22].

As in most registries, the procedural success rate in retrograde procedures in our study was lower (83.33%) than those involving an antegrade only approach (93.55%) and it was driven mainly by an increase in peri-procedural myocardial injury. Other factors that may lower the procedural success rate are the need for urgent repeat PCI or CABG, development of acute neurological deficit or an acute myocardial infarction during the hospital stay.

Having an 83.33% procedural success rate in retrograde procedures is a testament to the adequate training & experience of operators at our center as it is comparable to the results achieved in the European RECHARGE registry (86%) and the Japanese CTO PCI Expert Registry (88%) and higher than the results seen in the OPEN-CTO (74.7%), the PROGRESS-CTO (75.4%) and the EURO-CTO (75.3%) registries [16, 17, 23–26].

In a recent update from the PROGRESS-CTO registry antegrade-only cases, Pilot 200 (28%; Abbott Vascular) and Fielder XT (24%; Asahi Intecc) were the most commonly used guidewires, with a recent increase in the use of GLADIUS MONGO wire from 4 to 22% in the time period from 2020 to 2022 while Corsair (21%; Asahi Intecc) and Turnpike Spiral (20%; Vascular Solutions) were the most frequently used microcatheters. In retrograde cases, Sion (32%; Asahi Intecc) was the most frequent guidewire used, followed by Sion Black (22%; Asahi Intecc), Pilot 200 (22%), and Suoh 03 (19%; Asahi Intecc), while Corsair (16%) and Turnpike LP (11%) were the most commonly used microcatheters with a recent increase in the use of CORSAIR Pro XS [27].

In our study, the most commonly used guidewires in the antegrade cases were the GAIA 2nd (61.29%), GAIA 3rd (29.03%), CP12 (29.03%) and the Fielder XT (25.8%) while the most frequently used microcatheters was the Caravel (30.65%). The GLADIUS MONGO guidewire was only used in 7.5% of all cases due to its limited availability in our region despite its increased use internationally. In the retrograde group the most frequently used guidewires were the Sion Black (66.6%), Fielder XT-R (66.6%) and Sion (50%) while the most commonly used micro-catheter was the CORSAIR Pro in 72% of cases.

The retrograde group required longer procedure times than the antegrade group (136 min vs 92 min), larger volumes of contrast were used (Mean contrast volume 486 ml vs 338 ml) and higher exposure to radiation doses was found (Mean cumulative AK 9.8 Gy vs 6.4 Gy) which were statistically significant.

Regarding the retrograde procedures, the long procedure time was comparable to those seen in the Euro-CTO (159 min), the J-CTO (160 min), the PROGRESS-CTO (168 min) registries but higher than the procedure times seen in the OPEN-CTO (107 min) and the RECHARGE registries (90 min).

The average contrast volume used was much higher than those seen in Euro-CTO (387 ml), the J-CTO (241 ml), OPEN-CTO (278 min), PROGRESS-CTO (215 ml) and the RECHARGE registries (250 ml). This could be explained by a liberal use of contrast in our center with the under-utilization of IVUS (6.25% of procedures in our study) to guide the procedure due to the financial limitations limiting its availability and preventing its routine use in our cases.

In our study there was also a much higher average exposure to radiation doses compared to those seen in the OPEN-CTO (2.5 GY), the RECHARGE (1.6 GY) and PROGRESS-CTO (2.2 GY) registries. This could be explained by our extensive usage of cine, long fluoroscopy times, limited use of IVUS and not utilizing a reduced 7.5 FPS frame rate [16, 17, 24–26].

The in-hospital MACE rate is similar to results seen in the OPEN-CTO [15] registry (7%) but it is higher than MACE results seen in the PROGRESS-CTO (3.5%) [28], RECHARGE (2.6%) [16] and the LATAM registry (3%) [29]. This could be explained due to the relatively small number of patients in our study compared to these large international registries so the percentage of MACE in our study would appear larger than usual.

In-hospital mortality was 1.25% in our study which is similar to the registries previously mentioned and a testament to the very low levels of cardiovascular death associated with CTO procedures worldwide.

Three out of the 7 patients (42.86%) in our study who suffered from in-hospital MACE developed another Major adverse cardiac event during the 6-month follow-up period. This was a higher percentage than the patients who did not suffer from in-hospital MACE and was statistically significant.

In a recent meta-analysis, CTO patients undergoing PCI who developed peri-procedural myocardial injury faced a significantly higher risk of major adverse cardiac events, all-cause death, cardiac death, myocardial infarction, and target vessel revascularization during long-term follow-up which makes it an important marker for prognosis in these patients [30].

A recent study showed that multivessel artery disease, retrograde approach, and the presence of procedural complications were predictors of Peri-procedural myocardial injury (PMI) after CTO-PCI and that patients who develop PMI tend to have a poorer clinical prognosis and more MACE than those who do not develop PMI [31].

81.25% of patients in our study showed an improvement in the grade of their exertional dyspnea or angina within the 6-month follow-up period. The percentage of cases who showed improvement of symptoms after a 6-month period was similar between the 2 groups.

Several randomized clinical trials have yielded valuable insights into the effectiveness of CTO PCI. In the large DECISION-CTO trial where 834 patients were randomly assigned to either CTO or non-CTO PCI. The study revealed a high success rate of 90.6% for CTO PCI, but no significant differences were observed in the occurrence of death, MI, stroke, and target vessel revascularization between the two groups during the 4-year follow-up period and it did not report improved quality of life in the PCI arm [32].

However, careful examination of the study design and results reveals several limitations that need to be taken into consideration when interpreting the study results such as its underpowered nature due to early termination, a relatively high crossover rate from non-CTO PCI to CTO PCI (19%), a significant proportion of patients experiencing either no symptoms or mild symptoms, lack of information regarding the proportion of patients who underwent prior functional evaluation and its outcomes, and the study's non-inferiority design.

Other trials demonstrated enhancements in quality of life measures, but also did not exhibit significant differences in major adverse event rates such as the EURO-CTO trial where 396 patients were randomly assigned to CTO PCI or non-CTO PCI in a 2:1 ratio. After 12 months, patients who underwent CTO PCI achieved an 86% success rate, with notable improvements in angina frequency and quality of life. dimensions according to the Seattle Angina Questionnaire [33].

In a study conducted by Obedinskiy et al. (IMPACTOR-CTO trial) 94 patients with isolated right coronary artery chronic total occlusion (CTO) were randomly assigned to either CTO percutaneous coronary intervention (PCI) or non-CTO PCI at a single center. The results revealed that CTO PCI led to a greater reduction in ischemia and improvements in 6-min walk distance and quality of life, as assessed by the SF-36 health survey [34].

However, two other randomized clinical trials yielded different findings. One trial focused on patients with ST-segment elevation myocardial infarction (MI) (EXPLORE trial) assessing left ventricular systolic function and diastolic volume at 4 months. The other trial (REVASC trial) involved patients with stable angina evaluating segmental wall thickening in CTO territory and global and regional left ventricular function at 6 months [35, 36].

Both trials showed no significant differences between CTO PCI and non-CTO PCI in terms of hard outcomes such as MACE or improvement in left ventricular function. In contrast to the EXPLORE trial, REVASC showed a reduction in the combined clinical endpoint favoring CTO PCI, which was driven largely by a reduced need for clinically driven revascularization, with no difference in fatal outcomes [37].

A recent comprehensive meta-analysis was conducted, encompassing all existing prospective randomized and observational studies, to evaluate the correlation between successful CTO-PCI and patient life quality. The findings revealed a notable enhancement in SAQ scores among patients with CTO who underwent successful revascularization. Interestingly, a significant difference in scores was observed as early as 30 days after PCI and this improvement persisted throughout the longest follow-up period of 48 months. The results indicate that there is evidence to support the utilization of PCI as a treatment option for symptomatic patients with CTO who do not respond to medical treatment [38].

16.25% of patients in our study needed re-hospitalization within a 6-month period after PCI. The overall TLR rate at 6 months was 8.75%, the overall TVR rate at 6 months was 1.25%.

This was similar to the rates seen in the OPEN-CTO registry (14.8% re-hospitalization) [15] and in the EURO-CTO [19] with an average of 23 months of follow-up where re-hospitalization rate was 14.2% and the overall MACE including revascularization was 13.6%.

A study reviewing the one-year outcomes of the RECHARGE registry demonstrated a favorable overall MACE rate of 8% at 12 months post-discharge. Furthermore, similar to our study there was a low TLR rate (5.5%) and TVR rate (5.2%) seen 12 months after a technically successful procedure [39].

Our results are comparable with a large published study by Wilson et al. [40] who reported an 8.6% MACE rate, a TLR rate of 4.5% and a TVR rate of 5.0% 12 months after technically successful procedures. Previous reports from CTO cohorts report TLR of 6.3–10.7% with second generation DES [41, 42].

Regarding the effectiveness and safety of the antegrade vs retrograde approaches, a recent study by Eugene et al. [43] compared antegrade and retrograde approaches in 485 CTO patients where Procedural success for antegrade and retrograde was 94.4% and 84.6%, respectively. This was similar to success rates in our study. In-hospital MACE occurred in 19 (3.8%) CTO episodes and was more common in the retrograde group (6.6% vs 1.5%). The majority of these were myocardial infarction (17 of 19). There were nine coronary perforations, seven retrograde (3.1%), two antegrade (0.7%, *p* = 0.09), and one required pericardiocentesis (0.2%). Our study had a higher in-hospital MACE rate in the retrograde group (16.6%) compared to this study owing to the higher prevalence of peri-procedural Myocardial injury compared to the antegrade group while having no patients who required pericardiocentesis due to perforation.

Another recent study, the PROGRESS-MENATA registry demonstrated that high success and acceptable complication rates are currently achieved at experienced centers in the MENATA region using a combination of crossing strategies. Mean J-CTO score was 2.1 ± 1.2 and overall procedural success rates were high (92%) which was similar to our study. They had a low incidence (1.8%) of in-hospital major adverse cardiac events. Antegrade wire escalation was the most common crossing strategy used (followed by retrograde approach [44].

These high success rates and relatively low complication rates in multiple studies and registries further emphasize the increasing safety of these procedures when utilizing modern techniques and equipment.

Regarding the clinical implications of the results of our study, it should influence clinical practice in encouraging the development of CTO training programs in multiple centers across the country with the guidance of a proctor expert CTO operator to be able to help thousands of symptomatic CTO patients across the country with limited access to high quality care to be able to have a procedure done which is proven to improve their symptoms.

While maintaining high success rates and low complication rates, patient safety is ensured when carefully chosen patients with clear indications are operated upon under the guidance of expert operators in a well-equipped center and the stigma around the complication rates surrounding complex CTO procedures could be eventually decreased with increasing confidence in the success of CTO procedures.

Future research directions should be aiming to recruit a large number of patients across these multiple centers and aim to reproduce these results on a wider scale to be able to further emphasize the safety of CTO procedures when done in a carefully selected population under the guidance of expert operators.

### Study limitations

The current study was a prospective cohort study in which all study participants were recruited from a single center (Ain Shams University Hospitals) which may not be representative of the different centers in Egypt with a wide variability in CTO training programs and availability of high-volume CTO operators.

The implications regarding this is that not all PCI centers should be allowed to operate on complex CTO patients without having expert operators with a pre-defined yearly minimum number of patients operated on, while also having all the equipment needed for both crossing and also equipment to ensure bail-out and safety in case of occurrence of complications such as coils and graft stents. The results of this study should only be reciprocated and applied upon in centers with similar capabilities and operators with similar expertise in order to be able to generalize these results and reach success rates and safety from complications in similar rates.

A large sample size would help in better assessment of success rates and complication rates as well as recruiting larger number of patients in retrograde group for better accuracy in comparison with antegrade group. A longer follow-up period may help in detecting long-term MACE that may otherwise not be detected during the first 6 months.

The study did not evaluate the technical success rate between the different CTO techniques and did not compare short-term MACE between successful and failed CTO PCI procedures where patients continued on optimal medical therapy only.

## Conclusions

Our study was a single-center study experience from one of the highest volume centers of CTO PCI in Egypt. Retrograde procedures compared to antegrade were associated with longer procedure times, larger amounts of contrast used and higher exposure to radiation doses. Our results showed that technically successful CTO PCI in a well-equipped center with highly qualified CTO operators resulted in high procedural success rates and low incidence of short-term MACE.

## Data Availability

The datasets used and/or analyzed during the current study are available from the corresponding author on reasonable request.

## Abbreviations

CTO: Chronic total occlusion
TLR: Target lesion revascularization
PCI: Percutaneous coronary intervention
TVR: Target vessel revascularization
MACE: Major adverse cardiac events
CABG: Coronary artery bypass graft surgery
